# Supplementary material for: Current out of pocket care costs among HIV and hypertension co-morbid patients in urban and peri-urban Uganda
Source: PLOS Glob Public Health. 2024 Sep 25;4(9):e0003423. doi: 10.1371/journal.pgph.0003423 (PMC11423963; doi:10.1371/journal.pgph.0003423)
Supplement: S1 Appendix — (DOCX) [file pgph.0003423.s002.docx]

**S1 Appendix – GLM data procedures**

Instead of relying on a simple additive total of direct and indirect (and opportunity) costs per visit (as in outcomes a and b), we accounted separately in each outcome variable c and d for monthly hypertension drug costs. Survey instruments did not accurately account for individual input costs per visit for direct hypertension care (i.e., including line-items for medications, labs, procedures, visit fees, etc.) for more than half of the sample. Further, unlike direct HIV care costs where drugs are provided to patients for free, we have incomplete visit data on where, when, and how frequently patients in our sample purchase hypertension medications to manage their conditions. However, our survey does include a separate survey question which allowed us to estimate monthly spending on hypertension medications.

For outcomes c and d, we made the following adjustments to create final measures of monthly out of pocket spending on hypertension care. For respondents who reported no direct costs at last hypertension visit (n=30), we simply added stated monthly spending on hypertension drugs. For respondents for which we had complete input cost breakdowns for direct costs at last visit, including medications (n=45), we removed last visit medication costs and added stated monthly spending on medication data to the outcome variable. For the remaining patients who report a positive direct visit cost (n=19), but for whom we have no input cost breakdowns, we replace all direct visit costs with stated monthly medication spending costs. In this final category, one respondent reported no spending on medications, and we modified their final direct out of pocket spending to $0 USD. For the remaining 18 respondents, we assume that replacing last direct visit costs with monthly drug costs is likely to underestimate direct monthly expenditures on hypertension care by as much as 20-32% overall, based on patterns observed from patients with complete direct input cost breakdowns where the average share of direct care costs accounted for by medications ranges from 68-80%.

Finally, in all 4 GLM models, we impute missing values for reported household expenditure using median expenditure data from the whole sample. We tested various imputation methods including mean, median, OLS and GLM predicted household expenditure and find no significant departure from our main findings using any of these methods.
