# Supplementary material for: Current out of pocket care costs among HIV and hypertension co-morbid patients in urban and peri-urban Uganda
Source: PLOS Glob Public Health. 2024 Sep 25;4(9):e0003423. doi: 10.1371/journal.pgph.0003423 (PMC11423963; doi:10.1371/journal.pgph.0003423)
Supplement: S1 Table — (DOCX) [file pgph.0003423.s003.docx]

| **S1 Table. Mean cost (USD) per visit breakdown** | | | | | | | | |
| --- | --- | --- | --- | --- | --- | --- | --- | --- |
|  | Full Sample | |  | Full lockdown | | Partial lockdown | |  |
|  | Mean [SD] | n |  | Mean (SE) | n | Mean (SE) | n | p-value |
| HIV CARE |  |  |  |  |  |  |  |  |
|  |  |  |  |  |  |  |  |  |
| *Total direct cost* | $0.08 | 94 |  | $0.00 | 30 | $0.11 | 64 | 0.280 |
|  | [$0.48] |  |  | ($0.00) |  | ($0.07) |  |  |
|  |  |  |  |  |  |  |  |  |
| *Total indirect cost* | $4.80 | 94 |  | $2.89 | 30 | $5.69 | 64 | 0.005*** |
|  | [$4.60] |  |  | ($0.46) |  | ($0.64) |  |  |
|  |  |  |  |  |  |  |  |  |
| Transport costs (one-way) | $2.36 | 94 |  | $1.94 | 30 | $2.56 | 64 | 0.267 |
|  | [$2.52] |  |  | ($0.40) |  | ($0.33) |  |  |
| HH care costs | $0.36 | 94 |  | $0.39 | 30 | $0.35 | 64 | 0.841 |
|  | [$0.85] |  |  | ($0.16) |  | ($0.11) |  |  |
| Additional food expenses | $0.59 | 94 |  | $0.48 | 30 | $0.64 | 64 | 0.293 |
|  | [$0.70] |  |  | ($0.16) |  | ($0.08) |  |  |
| Other costs | $0.09 | 94 |  | $0.17 | 30 | $0.06 | 64 | 0.212 |
|  | [$0.39] |  |  | ($0.12) |  | ($0.02) |  |  |
|  |  |  |  |  |  |  |  |  |
| *Total opportunity cost* | $4.10 | 94 |  | $2.44 | 30 | $4.89 | 64 | 0.121 |
|  | [$7.13] |  |  | ($0.63) |  | ($1.03) |  |  |
|  |  |  |  |  |  |  |  |  |
| **Total (direct + indirect)** | **$4.87** | **94** |  | **$2.89** | **30** | **$5.81** | **64** | **0.004***** |
| **Total (direct + indirect + opportunity)** | **$8.98** | **94** |  | **$5.32** | **30** | **$10.69** | **64** | **0.009***** |
|  |  |  |  |  |  |  |  |  |

| **S1 Table. Mean cost (USD) per visit breakdown (cont'd)** | | | | | | | | |  |
| --- | --- | --- | --- | --- | --- | --- | --- | --- | --- |
|  | Full Sample | |  | Full lockdown | | Partial lockdown | |  |  |
|  | Mean [SD] | n |  | Mean (SE) | n | Mean (SE) | n | p-value |  |
|  |  |  |  |  |  |  |  |  |  |
| HTN CARE |  |  |  |  |  |  |  |  |  |
|  |  |  |  |  |  |  |  |  |  |
| *Total direct cost* | $9.82 | 94 |  | $4.40 | 30 | $12.36 | 64 | 0.053* |  |
|  | [$18.61] |  |  | ($2.00) |  | ($2.61) |  |  |  |
| *Total indirect cost* | $3.26 | 94 |  | $1.73 | 30 | $3.98 | 64 | 0.022** |  |
|  | [$4.46] |  |  | ($0.52) |  | ($0.61) |  |  |  |
|  |  |  |  |  |  |  |  |  |  |
| Transport costs (one-way) | $1.33 | 94 |  | $0.62 | 30 | $1.67 | 64 | 0.017** |  |
|  | [$1.99] |  |  | ($0.20) |  | ($0.28) |  |  |  |
| HH care costs | $0.24 | 94 |  | $0.19 | 30 | $0.26 | 64 | 0.652 |  |
|  | [$0.70] |  |  | ($0.14) |  | ($0.08) |  |  |  |
| Additional food expenses | $0.39 | 94 |  | $0.29 | 30 | $0.45 | 64 | 0.321 |  |
|  | [$0.73] |  |  | ($0.12) |  | ($0.10) |  |  |  |
| Other costs | $0.07 | 94 |  | $0.05 | 30 | $0.09 | 64 | 0.616 |  |
|  | [$0.34] |  |  | ($0.35) |  | ($0.05) |  |  |  |
|  |  |  |  |  |  |  |  |  |  |
| *Total opportunity cost* | $5.94 | 94 |  | $0.70 | 30 | $8.40 | 64 | 0.266 |  |
|  | [$31.13] |  |  | ($0.24) |  | ($4.69) |  |  |  |
|  |  |  |  |  |  |  |  |  |  |
| **Total (direct + indirect)** | **$13.08** | **94** |  | **$6.13** | **30** | **$16.34** | **64** | **0.026**** |  |
| **Total (direct + indirect + opportunity)** | **$19.02** | **94** |  | **$6.82** | **30** | **$24.74** | **64** | **0.043**** |  |
| **Notes:** ^$^ Though not individually reported, total direct costs of care for HIV and HTN included costs related to facility user fees, consultation fees, laboratory and test fees, and drug costs; Standard Deviations are in brackets; Standard Errors are in parenthesis; *** p<0.01, ** p<0.05, * p<0.1; Direct costs include facility user fees, consultation fees, laboratory and test fees, and drug costs; Other costs include cellular service fees, fines from law enforcement, and informal payments; Indirect cost totals and all financial and economic totals account for round-trip transportation costs. | | | | | | | | |  |
|  |  |  |  |  |  |  |  |  |  |
|  |  |  |  |  |  |  |  |  |  |
|  |  |  |  |  |  |  |  |  |  |
