# Supplementary material for: Current out of pocket care costs among HIV and hypertension co-morbid patients in urban and peri-urban Uganda
Source: PLOS Glob Public Health. 2024 Sep 25;4(9):e0003423. doi: 10.1371/journal.pgph.0003423 (PMC11423963; doi:10.1371/journal.pgph.0003423)
Supplement: S2 Table — (DOCX) [file pgph.0003423.s004.docx]

| **S2 Table. Mean monthly care costs, projected visits, and hospitalization costs** | | | | | | | | |  |
| --- | --- | --- | --- | --- | --- | --- | --- | --- | --- |
|  | Mean [SD] | n |  | *Financial costs* | |  | *Economic costs* | |  |
|  |  |  |  | Mean [SD] | n |  | Mean [SD] | n |  |
|  |  |  |  |  |  |  |  |  |  |
| *Monthly HIV care* |  |  |  |  |  |  |  |  |  |
| Projected number of monthly visits | 0.7 | 94 |  |  |  |  |  |  |  |
|  | [0.3] |  |  |  |  |  |  |  |  |
| Mean monthly total HIV care costs (USD) |  |  |  | $3.31 | 94 |  | $4.00 | 94 |  |
|  |  |  |  | [$3.53] |  |  | [$7.00] |  |  |
| Mean monthly HIV costs as % of HH income |  |  |  | 4.2% | 83 |  | 6.9% | 83 |  |
|  |  |  |  | [5.5%] |  |  | [8.6%] |  |  |
| Mean monthly HIV costs as % of HH expenditure |  |  |  | 5.1% | 82 |  | 8.0% | 82 |  |
|  |  |  |  | [8.3%] |  |  | [11.2%] |  |  |
| *Monthly HTN care* |  |  |  |  |  |  |  |  |  |
|  |  |  |  |  |  |  |  |  |  |
| Projected number of monthly visits | 1.5 | 94 |  |  |  |  |  |  |  |
|  | [1.8] |  |  |  |  |  |  |  |  |
| Drug costs (USD) | $7.41 | 92 |  |  |  |  |  |  |  |
|  | [$10.13] |  |  |  |  |  |  |  |  |
| Mean monthly total HTN care costs (USD) |  |  |  | $14.03 | 94 |  | $22.94 | 94 |  |
|  |  |  |  | [$18.79] |  |  | [$53.48] |  |  |
| HTN costs as % of HH income |  |  |  | 16.9% | 83 |  | 22.4% | 83 |  |
|  |  |  |  | [26.9%] |  |  | [38.4%] |  |  |
| HTN costs as % of HH expenditure |  |  |  | 20.1% | 82 |  | 27.1% | 82 |  |
|  |  |  |  | [49.0%] |  |  | [65.2%] |  |  |
|  |  |  |  |  |  |  |  |  |  |
| *Hospitalization (HTN)* |  |  |  |  |  |  |  |  |  |
|  |  |  |  |  |  |  |  |  |  |
| % hospitalized for HTN in last 12 months | 9.6% | 94 |  |  |  |  |  |  |  |
| Number of hospital stays in last 12 months | 1.4 | 9 |  |  |  |  |  |  |  |
|  | [0.9] |  |  |  |  |  |  |  |  |
| Per hospitalization stay cost (USD) | $124.77 | 9 |  |  |  |  |  |  |  |
|  | [$181.43] |  |  |  |  |  |  |  |  |
| Notes: "Economic costs" category (col 3) includes foregone wage earnings due to HIV or HTN visit. "Financial cost" totals do not include foregone wage earnings from medical visits; Standard deviations reported in brackets; Monthly HTN care costs include hospitalization and drug costs, monthly HIV care costs do not. | | | | | | | | |  |
|  |  |  |  |  |  |  |  |  |  |
|  |  |  |  |  |  |  |  |  |  |
