# Supplementary material for: Current out of pocket care costs among HIV and hypertension co-morbid patients in urban and peri-urban Uganda
Source: PLOS Glob Public Health. 2024 Sep 25;4(9):e0003423. doi: 10.1371/journal.pgph.0003423 (PMC11423963; doi:10.1371/journal.pgph.0003423)
Supplement: Attachment — Submitted filename: Response to Reviewers.docx [file pgph.0003423.s005.docx]

**Reviewer Responses**

PGPH-D-24-00258

Out of Pocket Care Costs Among HIV and Hypertension Co-morbid Patients in Urban and Peri-Urban Uganda

*Journal Requirement:*

| Comment | Response |
| --- | --- |
| 1. Please include a complete copy of PLOS’ questionnaire on inclusivity in global research in your revised manuscript. Our policy for research in this area aims to improve transparency in the reporting of research performed outside of researchers’ own country or community. The policy applies to researchers who have travelled to a different country to conduct research, research with Indigenous populations or their lands, and research on cultural artefacts. The questionnaire can also be requested at the journal’s discretion for any other submissions, even if these conditions are not met. Please find more information on the policy and a link to download a blank copy of the questionnaire here: https://journals.plos.org/globalpublichealth/s/best-practices-in-research-reporting. Please upload a completed version of your questionnaire as Supporting Information when you resubmit your manuscript. | Thank you for providing a link to the policy and questionnaire. We have attached a completed S1 Checklist to our submission and have included a sentence referencing the checklist in the Method section of the main manuscript on page 12 lines 244-46:  “*Inclusivity in global research*  Additional information regarding the ethical, cultural, and scientific considerations specific to inclusivity in global research is included in the Supporting Information (S1 Checklist).” |

Reviewer #1: Overall comments:

| Comment | Response |
| --- | --- |
| This study measures the out-of-pocket care costs among patients diagnosed with HIV and Hypertension in Urban and Peri-urban settings of Uganda. The study addresses an important topic and fill the knowledge gap in this area; however, there are several aspects that could be clarified or strengthened throughout the paper. Therefore, I have made several specific comments and hope this will help the authors improve this paper's quality. | We are appreciative of the close and careful reading from Reviewer 1 and the constructive comments provided to improve the manuscript. Thank you for taking the time to assess our work and provide this feedback. |

Specific comments:

| *Comment* | *Response* |
| --- | --- |
| Title:  1. The authors identified the type of costs (i.e., out of pocket care expenses) in the title, however it is not clear if these cost estimations were done retrospectively or focused a current state of illnesses (i.e., snapshot). It would be good to highlight this in the title, so readers get it at the very outset of the paper. | Thank you for this comment. We have revised the title as follows:  *“Current out of pocket care costs among HIV and hypertension co-morbid patients in urban and peri-urban Uganda”*  We have also revised the manuscript abstract on page 2 lines 37-8 with the following:  *“This study aims to build evidence on the burden of current out-of-pocket costs of care among HIV-HTN co-morbid patients.”* |
| Abstract:  1. The authors need to add a primary "study objective” statement in the background. | We have added the following (page 2 lines 37-8):  *“This study aims to build evidence on the burden of current out-of-pocket costs of care among HIV-HTN co-morbid patients.”* |
| 2. The authors stated that respondents were sampled during the nationwide lockdown, but it is not clear what is the sampling cohort/frame of this study (for example, how many potential patients HIV/HTN are seen in 10 clinics on an average in a year)? | Thank you for this comment. We have both revised and added more information to the following sentence (page 6 lines 147-9):  *“At the time of study planning, we did not know how many patients per facility were co-morbid (with HIV and HTN). Sample size per facility was determined based on estimated HTN prevalence within the population [14].”*  We have also added the following line on page 7 lines 159-63:  *“All facilities were proportionally represented in the study based on the total number of HIV patients that was known at the time of study planning, with around 0.2 percent of each population of PLHIV (who were also co-morbid with HTN) from each clinic included in the study. As this study was exploratory / hypothesis generating, there were no ex-ante power calculations conducted.”* |
| Introduction:  1.In line # 66 on Pg3, the authors need to provide the full forms for “HIV”, and “UNAIDS”. | We have revised the manuscript as follows on page 3 lines 65-67:  *“As of 2021, according to the Joint United Nations Programme on HIV/AIDS (UNAIDS), 89% of people living with HIV (PLHIV) in Uganda knew their status, 92% of those received antiretroviral therapy, and 95% of those had achieved viral suppression [2].”*  However, we feel that the term HIV is used broadly enough in this publication and elsewhere so as to negate the need to provide full form of the acronym. |
| 2. I wonder if the term “PLHIV” is widely known (cited or common) for readers in this journal? If not, it may be better to use just HIV patients. | Thank you for this comment. After reviewing previous editions of this journal, we find that the term “PLHIV” appears to be common in PLOS Global Public Health and other health literature (especially in HIV literature). We have therefore kept the term in the manuscript but ensured it is defined early, see page 3 lines 65-67:  *“As of 2021, according to the Joint United Nations Programme on HIV/AIDS (UNAIDS), 89% of people living with HIV (PLHIV) in Uganda knew their status, 92% of those received antiretroviral therapy, and 95% of those had achieved viral suppression [2].”* |
| 3. It seems like the PULESA-Uganda trial will take place in 16 facilities, however this paper reports on the survey undertaken in 10 clinics. Could this be clarified in the intro. or the methods section, if possible? | Thank you, we have made the following changes on page 6 lines 135-8:  *“After stratifying clinics by relative size and type across the two districts, we selected ten facilities (five each in Wakiso and Kampala districts) in which to administer the formative assessments (see Table 1). A different combination of 16 clinics was later chosen for inclusion in the PULESA-Uganda trial.”* |
| 4.The rationale of this study in connection to the PULESA-Uganda trial could be misleading and is not supported by the evidence. In line # 111 on Pg5, the authors reported that findings from this cross-sectional survey will help to understand the potential efficiencies that might result from the integration. I find this statement problematic because the trial has not started yet, and how could authors make a claim that trial will result in fewer visits or improve efficiencies? It could be that trial findings are not statistically in favor to support this claim (or hypothesis). What if the trial finds more frequent visits than initially hypothesized? Anyway, I think this part needs to be reframed in a way that only focuses on understanding the costs, which is an important aspect to inform the subsequent economic model design and input parameters for the trial. | Thank you for this comment. We agree and have struck much of the language describing the main trial as suggested to alleviate confusion while reframing the section on page 4 lines 88-103 as follows:  *“One of the aims of the PULESA-Uganda trial is to evaluate two combinations of strategies to integrate care for HIV and HTN. This survey collected data on access to and the costs of HIV and HTN care prior to formal integration of HIV and HTN care through the PULESA-Uganda trial, and so provides insight into potential inefficiencies in care access and cost that may have been present prior to integration of these services. The current perception is that most comorbid patients manage their HIV and HTN at different clinics and separate clinical visits, though in practice little is known about current care seeking behavior. Current literature suggests that HTN treatment initiation, 1-year retention, and control are suboptimal among co-morbid patients and demonstrate notable quality gaps [4, 5]. Co-locating and/or synchronizing service delivery for multiple conditions can theoretically reduce the number of required clinic visits, creating efficiencies that manifest as savings in health-related patient expenditures. Results of this survey would help to inform the future testing of such a hypothesis. These results would also inform the subsequent economic model design and input parameters for the trial.*  *We provide the results of this pilot out-of-pocket survey to gain insight into the financial and economic burden on PLHIV and HTN and to understand the potential efficiencies and inefficiencies that might result from care integration.”* |
| 5. The authors need to add more literature on the context of HIV and HTN burden and care seeking practices in the introduction. At this point, it seems like they only provided information about the trial and how this study will link to it. The readers do not know much about the context of care seeking practices, so I would like to see that added to the intro. section. | Thank you for this comment. We have removed several paragraphs of information about the trial which we deemed superfluous and have included the following regarding care seeking practices for comorbid HIV and HTN patients in Uganda. Please see the revisions on page 4 lines 88-98:  *“One of the aims of the PULESA-Uganda trial is to evaluate various strategies to integrate care for HIV and HTN. This survey collected data on access to and the costs of HIV and HTN care prior to formal integration of HIV and HTN care through the PULESA-Uganda trial, and so provides insight into potential inefficiencies in care access and cost that may have been present prior to integration of these services. The current perception is that most comorbid patients manage their HIV and HTN at different clinics and separate clinical visits, though in practice little is known about current care seeking behavior. Current literature suggests that HTN treatment initiation, 1-year retention, and control are suboptimal among co-morbid patients and demonstrate notable quality gaps [4,5]. Co-locating and/or synchronizing service delivery for multiple conditions can theoretically reduce the number of required clinic visits, creating efficiencies that manifest as savings in health-related patient expenditures.”* |
| Methods:  1. The authors did not use the standard format for reporting the economic evaluation. It is advisable to use (and cite) the CHEERs checklist in this paper (please see: <https://www.ispor.org/heor-resources/good-practices/article/consolidated-health-economic-evaluation-reporting-standards-(cheers)---explanation-and-elaboration>) | Thank you for this comment. We agree and have added a CHEERs checklist to the supplementary files. We note this in the current manuscript on page 5 lines 109-11:  *“The main reporting elements, as outlined in the Consolidated Health Economic Evaluation Reporting Standards (CHEERS) checklist, can be found in the Supplemental Information (S2 CHEERS Checklist)[12].”* |
| 2. In line # 121 on page 5, the authors mentioned that survey was adapted from a previous study. Upon checking the reference (citation 17), it seems like this study reported on the financial costs of HIV, so it is not clear what aspects of the tool were adapted and what areas were added to the adapted version? | Thank you for this comment. We have revised the selected passages as follows. See page 5 lines 115-22:  *“The OOP survey was adapted from a survey developed previously for use in Cote d’Ivoire and consisted of a pre-tested questionnaire to estimate and describe the out-of-pocket cost expenditures of PLHIV and HTN [13]. The original survey collected only financial out of pocket costs and was adapted significantly for the local context in Uganda and to capture a more expansive set of economic out of pocket costs faced by patients. The survey contained questions on each person’s most recent medical visits for each condition (for HIV, the most recent visit was that day’s visit; for HTN, patients were asked to recall their last visit for care, which took place before the date of the survey within the last 12 months).”* |
| 3. in line # 125 on page 5, could you please clarify what was the time frame for the recall? How did you define the last visit for care? Was that in the last year, two years, etc. from the date of survey? Please clarify the time point. | We have added clarifying language to the referenced section see page 5 lines 120-22:  *“The survey contained questions on each person’s most recent medical visits for each condition (for HIV, the most recent visit was that day’s visit; for HTN, patients were asked to recall their last visit for care, which took place before the date of the survey within the last 12 months).”* |
| 4. In line # 131 on page 6, the authors reported that facilities > 2000 patients were classified as large facilities. What is the basis for this criterion? Is that something based on the standard of care or assumption (please clarify)? | We have modified language around facility size and sampling as follows (see page 6 lines 127-38):  *“We purposefully selected a sample of clinics to conduct formative research in advance of the PULESA-Uganda trial. In planning our project, we obtained a comprehensive list of all HIV clinics in both Wakiso and Kampala districts. After review, we determined that clinics serving fewer than 400 patients per year would be heterogenous and difficult to study. Among the remaining 60+ clinics serving more than 400 patients, the median clinic size was roughly 2,000 patients per year. We sought to include an equal number of small (<2,000 patients) vs. large (>2,000 patients) clinics so that study findings could be more generalizable. Further, we believed that the larger HIV clinics would be more likely to have resources not available to smaller ones. We also aimed to include a mix of public and private/not-for-profit facilities. After stratifying clinics by relative size and type across the two districts, we selected ten facilities (five each in Wakiso and Kampala districts) in which to administer the formative assessments (see Table 1). A different combination of 16 clinics was later chosen for inclusion in the PULESA-Uganda trial.”* |
| 5. Table 1, It seems like all small facilities (<2000 patients) represent the public sector facilities.  I wonder, if you could reclassify the small facility size in way, say for instance, <3000 patients, you could have one small facility representation in the PNFP sector. Just a thought! | Thank you for this comment. Notably, aside from mentioning it in our sampling strategy, we do not include size or type in any of our analyses. We have included the following additional description regarding facility size and type on page 12 lines 233-43:  *“For models 1-4 we carefully considered the inclusion of additional clinic-level covariates such as facility size and facility type (public vs. private/not-for-profit). In our HIV models (1 and 2) direct HIV costs that would be realized at a given facility constituted a vanishingly small share of total costs of care, insufficient in our estimation to justify the inclusion of additional descriptive covariates for these facilities. Further, we do not have reason to assume that characteristics like patient load or facility type would have any relationship to the predominately indirect and opportunity costs of HIV care included in the dependent variable beyond what should be captured by including a vector (γ_c) of facility fixed effects. Thus, these variables were not included to avoid concerns about model over-fit. In models 3 and 4 we did not have any data on the HTN facilities themselves visited for HTN care to include additional facility-level covariates such as clinic size or type.”* |
| 6. In line 145 on page 5, there is mention of sample size based on HTN prevalence, but I do not see any calculations or formulas showing how this was determined? Authors must provide the sample size calculation to demonstrate that 94 participants represent adequate power or precision. | Thank you for your comment. We have revised the manuscript to provide clarity on the sample size. Please see page 7 lines 159-63:  *“All facilities were proportionally represented in the study based on the total number of HIV patients that was known at the time of study planning, with roughly 0.2 percent of each population of PLHIV (who were also co-morbid with HTN) from each clinic included in the study. As this study was exploratory / hypothesis generating, there were no ex-ante power calculations conducted.”* |
| 7. The costs are reported as of 2022, so it would be good to use the World Bank’s GDP index of 2023/24 considering the readers may want to know the costs as of the current year. | Thank you for your comment. Unfortunately, final 2023 (and 2024) data were not available from the World Bank Implicit Price Deflator at the time of response to this comment, only rates by quarter (excluding Q4). |
| 8. In the analysis, the authors reported the application of generalized linear model. I think, there may be a clustering by the type and size of clinic, as some patients may be paying more or less depending on where they are treated. In this case, my suspicion is that clinic type/size may be playing a random effect modifier on the out of pocket costs. Therefore, I wonder if the authors could consider applying the generalized linear Mixed-Effect model (GLMM) instead? | Thank you for your suggestion. We have carefully considered the inclusion of clinic type and clinic size covariates as well as the model specification (GLMM vs GLM) and functional form. We have included the following explanation on page 12 lines 233-43 to address this concern more directly:  “*For models 1-4 we carefully considered the inclusion of additional clinic-level covariates such as facility size and facility type (public vs. private/not-for-profit). In our HIV models (1 and 2) direct HIV costs that would be realized at a given facility constituted a vanishingly small share of total costs of care faced by patients, which was insufficient in our estimation to justify the inclusion of additional descriptive covariates for these facilities. Further, we do not have reason to assume that characteristics like patient load or facility type would have any relationship to the predominately indirect and opportunity costs of HIV care included in the dependent variable beyond what should be captured by including a vector (*$\gamma_{c}$*) of facility fixed effects. Thus, these variables were not included to avoid concerns about model over-fit. In models 3 and 4 we did not have any data on the HTN facilities themselves visited for HTN care to include additional facility-level covariates such as clinic size or type.”*  We have also slightly modified each of our regression models on pages 10-11 lines 188-232 to reflect the inclusion of facility fixed effects ($\gamma_{c}$) to account for any differences at the cluster level, and re-run our main models to include fixed effects, making needed changes to our findings and discussion.  Finally, we discuss the use of GLMM as a robustness check on pages 26-7 lines 389-93:   *“As a robustness check, we additionally tested the use of a generalized linear mixed-effects (GLMM) specifications for each of the 4 models, treating each of the clinics as a random effect modifier. Our results did not differ meaningfully from those reported in Tables 5 and 6.”* |
| 11. Considering that costs were changing (between full and partial lockdowns), could the authors perform a sensitivity analysis to demonstrate how robust their cost estimates can be when assuming a 10%, 15% or 25% etc. variation in cost over time scenario? | Thank you for this comment. This is a great idea, but this paper might not be well-suited for this type of analysis. Though we do control for whether patients were sampled during or after full lockdown, our data are not time-series. We have included the following language in the limitations section to address this suggestion, see page 38 lines 604-8:  *“Finally, this analysis is limited in that we utilize cross-sectional data to report on costs at a given moment in time for patients and, given our small sample sizes and limited set of covariates, we do not predict cost estimates through our regression analyses to inform policy. Instead, our analyses should be seen as exploratory and hypothesis generating.”* |
| Results:  1. In Table 2, could authors relabel the variable in column three “No lockdown” to “partial lockdown” based on how they defined in the methods section? | Thank you for this comment. The heading in Table 2 is now relabeled to “partial lockdown”, see pages 14-15.  We have also re-labeled corresponding column in Table 3 to also reflect this description from the methods section and used consistent terminology throughout the manuscript to differentiate between the full lockdown period of 42 days, and the “partial lockdown” period that followed and struck all reference to “no lockdown”. |
| 2. In Tables 2 and 3, considering the sample size in each lockdown and partial lockdowns are small, could authors report the estimates in median (and interquartile ranges-IQR) instead? It seems that standard error of means is flagging the need for medians and IQRs. | Thank you for this question. We had accidentally labeled reporting 95% Cis in Table 4 and have changed this error to reflect the inclusion of median and IQR values in Table 4. Please see page 21.  We have modified Table 3 as suggested to report median costs and IQRs (see pages 18-19). We have also included mean tables 3 and 4 as Appendix Tables A1 and A2 to account for the mean comparison tests run in those tables and as a point of reference for the reader, as many median input costs are reported as $0.00.  We considered the request for Table 2 but determined that as most of Table 2 reports descriptive statistics (and not costs), reporting median values is not informative and eliminates our analysis of mean comparisons between our lockdown and no lockdown sample. We have kept Table 2 as a mean comparison table. |
| 3. In Table 6, did authors consider a interaction term for variables ‘travel time to/from HTN facility’ and ‘admitted overnight’? It could be that patients who live far (in rural remote areas) had to stay in the hospital for an extra night or so, just because discharging them early was not feasible or they may have to return back due to disease complications etc. | Thank you for this comment. We have addressed this in the limitations section of the manuscript on pages 37-8, lines 597-604:  *“Seventh, some of the large input costs of care we observe from hospitalization for either HIV- or HTN-related conditions may be in part due to patients who live far from these facilities (in remote rural areas) and require overnight hospital care because being discharged early was not feasible, or imminent returns to the facility for follow-up care or complications would be necessary. Unfortunately, we cannot evaluate this hypothesis as our data on the facilities where patients were hospitalized for either condition is incomplete. It may not be reasonable to assume that the same facilities at which patients receive regular (or their last) HTN or HIV care would be the same ones utilized for overnight hospitalization.”* |
| Discussion:  1. Although it is understandable that fewer visits during the full lockdown may have contributed to lower costs, I wonder if there was any change in how patients sought care that may be cheaper than the traditional ways of seeking care? For example, online/virtual consultation with the care provider may be low cost, and prevented time/travel and missed opportunities? Could authors reflect on this in the discussion depending on the context of care in Uganda? | Thank you for this comment. We have revised the limitations section of the manuscript as follows on pages 36-7, lines 576-82:  *“We also did not observe any data on changes in how patients sought care during full lockdown that may be cheaper than traditional modes (e.g., shifting to online or virtual consultations). Such a shift may have led to a more optimal decrease in care costs that might not correspond with a resulting decrease in care quality, which we would not capture. Therefore, the out-of-pocket patient costs reported here may over- or (more likely) under-represent the true cost of proper disease management of either condition under full compliance of recommended treatment and/or may omit other important medical factors.”* |
| 2. The section on “limitations” could be reworded as “Strengths and Limitations” to better align with the content in this section. | The section has been reworded accordingly in the revised document. The section title now reads:   “Strengths and Limitations” (see page 35 line 558). |
| 3. I think the statement (in line # 494), where authors report “while potential savings from integrating care at HIV……..of either condition” should be omitted or rephrased because it is not supported by any literature and the future trial findings cannot be assumed at this stage. | We agree and have omitted this claim in the revised document. The line “while potential savings from integrating care at HIV… of either condition” has now been struck from the Strengths and Limitations section. |

*Reviewer #2:*

| *Comment* | *Response* |
| --- | --- |
| The manuscript is methodologically sound, with the data backing the conclusions. All data supporting the findings in the manuscript has been thoroughly provided. Authors are advised to enhance readability by revising the language | Thank you for your comments. We have adjusted the language in the main manuscript to enhance readability throughout. |

Thank you for these comments. In addition to the changes outlined above, we have included a few additional changes:

- Title has been modified to sentence case.
- We modified our limitations to reflect further considerations about generalizability (see page 36 lines 565-72):

*“First, compared to other studies of out-of-pocket costs for HIV and HTN in sub–Saharan Africa, our sample population is relatively small and relies exclusively on patient self-report, which adds both imprecision and potential bias to our cost estimates. Second, though our patient population may be proportional to the number of co-morbid patients enrolled in facilities from which they were sampled and represent a decent cross section of medium-to-large HIV facilities (i.e., those serving >400 patients per year) in the country, we caution that the subset of patients who took the OOP survey were drawn from a larger convenience sample of patients selected for the KAP survey.”*

- As mentioned in the text above, we have included facility fixed-effects in all 4 of our regression models which required updating both regression tables and results and discussion sections accordingly.
- In the process of adding facility fixed effects to all models (and exploring facility type and facility size), as suggested by reviewer 1, we discovered that we had access to information on whether sampled patients attended the same HIV facilities where they were sampled for this survey for HTN care (at last HTN visit). This variable seemed critical to include in HTN models and is now included in models 3 and 4 and is discussed on page 11 lines 225-7:

*“However, we do include an additional binary covariate same facility to indicate whether the patient self-reports attending the same HIV clinic for their HTN care versus a different facility/location.”*

We also discuss basic descriptive breakdowns of this variable in our findings section on pages 19-20 lines 314-326:

*“Though not reported in Table 3, we also examine the total per-visit hypertension care costs according to whether patients reported visiting the same HIV facility for their last HTN care visit (n=24) versus a different HTN facility (n=70) for both financial (direct + indirect) and economic (financial + opportunity) costs. We find that for financial costs, the median per-visit cost for HTN care among those who report using the same HIV facility is $7.09 (IQR: $4.24-$10.43) while those using different facilities report a per-visit cost of $5.26 (IQR: $1.46-$18.71). For economic costs, the median per-visit cost for HTN care among those who report using the same HIV facility is $8.40 (IQR: $4.97-$11.98) while those using different facilities report a per-visit cost of $6.28 (IQR: $1.46-$23.38). Using student’s t-test, we find that mean differences are flipped (same-facility visits are cheaper on average than different facility visits) but that neither mean difference is statistically significant. Further, we lack sufficient data to examine whether patients who reported using the same HIV facilities for their HTN care combined any of their care visits with HIV appointments.”*

We also discuss this in our findings section on page 26 lines 372-5:

*“We also find that number of monthly care visits, travel time to facilities, and whether patients visit different HTN facilities from the HIV clinics at which they were sampled all show a positive relationship with out-of-pocket spending (again, significant in three out of four model specifications).”*

Finally, we discuss this in our discussion section on page 32 lines 482-97:

*“Importantly, we do find evidence that a small proportion of co-morbid patients are already engaging in some form of HTN care at some of the study’s HIV facilities during our pilot. We do not find a significant difference in per-visit costs of HTN care among those who reported visiting the same HIV facilities for their HTN care compared to those reporting the use of different facilities. However, data to support this conclusion are incomplete – the small total number of patients (n=24) and wide range of costs incurred increase the uncertainty of our estimates – and little is known about the overall quality or extent of care received at HIV facilities prior to a formal integration of services (i.e., whether “care” includes only BP screening/monitoring or actual treatment). Limited published evidence suggests that some screening/monitoring is already taking place at many HIV facilities across sub-Saharan Africa, but both screening and treatment are infrequently observed [47]. Compellingly, in our final regression models, we do find a consistently positive (and mostly significant) relationship between attending different facilities for HTN care than those where HIV care is sought and the monthly cost of HTN care. However, these findings do not include HIV costs nor reflect any adjustments for bundling care for different visit types. More evidence on clinically-ideal integration of services and the current standard of care is urgently needed.”*

- Finally, we also modified the number of decimal places reported in several tables to reduce clutter and facilitate easier reading of the tables.
